# “Giant tick” attacks: dynamics of *Hyalomma lusitanicum* and detection of *Rickettsia sibirica mongolitimonae* in southern France

**DOI:** 10.1051/parasite/2026037

**Published:** 2026-07-09

**Authors:** Philippe Parola, Bouthaina Hasnaoui, Basma Ouarti, Zaina Amirat, Rym Bouledroua, Lionel Almeras, Elodie Gerbeau, Noelle Masotti, Jean-Michel Bérenger, Frederic Stachurski, Adama Zan Diarra

**Affiliations:** 1 Aix Marseille Univ, SSA, RITMES Marseille France; 2 IHU Méditerranée Infection Marseille France; 3 Centre de référence (Sud) des maladies vectorielles liées aux tiques, AP-HM Marseille France; 4 Unité Parasitologie Entomologie, Département Microbiologie et Maladies Infectieuses, Institut de Recherche Biomédicale des Armées 13005 Marseille France; 5 Service Mer, Milieux Aquatiques et Zones Humides Direction, Pilotage du Grand Cycle de l'Eau, DGD Transition Environnementale, Eau, Culture et Sport, Aix Marseille Provence Métropole Marseille France; 6 CIRAD, UMR ASTRE Montpellier France; 7 ASTRE, Univ Montpellier, CIRAD, INRAE Montpellier France; 8 Institut de recherche pour le développement (IRD), Maladies Infectieuses, Négligées et Émergentes au Sud (MINES) Marseille France

**Keywords:** Ticks, *Hyalomma lusitanicum*, *Rhipicephalus pusillus*, *Ixodes ventalloi*, *Haemaphysalis hispanica*, *Rickettsia sibirica mongolitimonae*

## Abstract

Since 2019, media reports have raised concerns about the emergence of “giant ticks” in Europe, particularly *Hyalomma marginatum*, due to its potential to transmit the Crimean-Congo haemorrhagic fever virus (CCHFV). In 2022, following several reports of unusually large ticks displaying aggressive behaviour toward humans in the Étang de Bolmon area (Bouches-du-Rhône, southern France), a popular area for walking and nature exploration, an investigation was conducted to characterise the local tick fauna, their seasonal activity, and associated microorganisms. Between 2022 and 2025, a total of 2,940 ticks were collected through environmental and host-based sampling, with 89% obtained from the ground and 11% from rabbits. Morphological, molecular, and MALDI-TOF mass spectrometry analyses were carried out and four species were identified: *Hyalomma lusitanicum* (78%), *Rhipicephalus pusillus* (22%), *Haemaphysalis hispanica* (<1%), and *Ixodes ventalloi* (<1%). The “giant ticks” were identified as *H. lusitanicum*, not *H. marginatum*. Molecular screening revealed *Rickettsia sibirica mongolitimonae* in *H. lusitanicum*, suggesting its role in the ecology of this emerging human pathogen, along with the endosymbiont *Candidatus* Midichloria mitochondrii. This work also confirmed the accuracy of MALDI-TOF MS as a reliable tool for rapid tick identification and highlights the re-emergence of *H. lusitanicum* in southern France, while indicating that the associated risk to human public health currently remains relatively limited.

## Abstract (fr)

Depuis 2019, les médias ont fait part de leur inquiétude quant à l’émergence de « tiques géantes » en Europe, notamment *Hyalomma marginatum*, en raison de son potentiel de transmission du virus de la fièvre hémorragique de Crimée-Congo (VFHCC). En 2022, suite à plusieurs signalements de tiques de taille inhabituelle présentant un comportement agressif envers l’homme dans la région de l’Étang de Bolmon (Bouches-du-Rhône, sud de la France), un site prisé pour la randonnée et la découverte de la nature, une étude a été menée afin de caractériser la faune locale de tiques, son activité saisonnière et les micro-organismes associés. Entre 2022 et 2025, 2 940 tiques ont été collectées par échantillonnage environnemental et sur leurs hôtes, dont 89 % prélevées au sol et 11 % sur des lapins. Les analyses morphologiques, moléculaires et par spectrométrie de masse MALDI-TOF ont permis d'identifier quatre espèces : *Hyalomma lusitanicum* (78 %), *Rhipicephalus pusillus* (22 %), *Haemaphysalis hispanica* (< 1 %) et *Ixodes ventalloi* (< 1 %). Les « tiques géantes » ont été identifiées comme *H. lusitanicum*, et non *H. marginatum*. Le criblage moléculaire a révélé la présence de *Rickettsia sibirica mongolitimonae* chez *H. lusitanicum*, suggérant son rôle dans l'écologie de ce pathogène humain émergent. L'endosymbiote Candidatus Midichloria mitochondrii a également été detecté. Ces travaux ont également confirmé la fiabilité de la spectrométrie de masse MALDI-TOF pour l'identification rapide des tiques et ont mis en évidence la réémergence de *H. lusitanicum* dans le sud de la France, tout en indiquant que le risque associé pour la santé publique reste actuellement relativement limité.

## Introduction

Ticks are obligate, blood-feeding, temporary ectoparasitic arthropods belonging to the subclass Acari and the order Ixodida*.* They are divided into two main families: Ixodidae (hard ticks), comprising over 700 species worldwide, and Argasidae (soft ticks), comprising roughly 200 species [5]. Ticks feed on mammals, birds, and reptiles, and many species may also bite and feed on humans. Moreover, ticks can act as vectors by efficiently transmitting some of the microorganisms they carry. Importantly, they harbor a vast range of microorganisms, including bacteria, viruses, and protozoa, some of which are pathogenic to humans and other animals [23].

In Europe, the tick genera most frequently implicated in human infectious diseases are *Ixodes*, *Rhipicephalus*, and *Dermacentor* spp. [30]. In France, four tick species are currently considered of medical importance. *Ixodes ricinus* is the main vector of spirochetes belonging to the *Borrelia burgdorferi* sensu lato complex responsible for Lyme disease. It may also harbor several other tick-borne bacterial agents, including *Rickettsia* spp., and is a vector of tick-borne encephalitis virus in north-eastern France. *Dermacentor marginatus*, the ornate sheep tick, and *Dermacentor reticulatus*, the ornate dog tick, are recognized vectors of spotted fever group (SFG) rickettsioses. *Rhipicephalus sanguineus sensu stricto*, the brown dog tick, is known as the vector of *Rickettsia conorii conorii*, the causative agent of potentially life-threatening Mediterranean spotted fever in southern France. *Rhipicephalus sanguineus* is also known to harbor other rickettsial organisms, including human pathogens [35, 47, 50].

More recently, *Hyalomma marginatum* has attracted growing attention in France. Until recent years, continental France was considered free of this large tick species; however, the presence of reproducing populations of *H. marginatum* in parts of southern France has now been demonstrated [3]. This tick is known to harbor several human pathogens, including the Crimean-Congo hemorrhagic fever virus (CCHFV), and has therefore become a focus of increased investigation in France [1, 3, 4, 32].

Since 2019, several media outlets have raised public concern regarding the emergence and potential spread of “giant ticks” (*H. marginatum*) in Europe, including in France [11]. In 2022, as a national reference center for tick-borne diseases, we were alerted by local walkers to the presence of unusually large and aggressive ticks in a specific area of southern France (Étang de Bolmon, Bouches-du-Rhône) and decided to investigate the exact location where these encounters occurred.

The primary objective of this study was to collect and identify ticks in this area and to assess their annual dynamics. Secondary objectives included further assessing matrix-assisted laser desorption/ionization time-of-flight mass spectrometry (MALDI-TOF MS) for tick identification and detecting specific microorganisms carried by these ticks using molecular tools.

## Material and methods

### Ethics

Management of the site was transferred in 2017 to the Aix-Marseille Provence Métropole, the large intermunicipal authority (a “metropolis” in the French administrative system) that governs the wider Marseille region in southern France [13]. Investigations and research activities were conducted under the supervision of the Sea, Aquatic Environments and Wetlands Department within the Directorate for Integrated Water Cycle Management. Ferreting operations are authorized through an application that hunters must submit each year to the Coastal Protection Agency (Conservatoire du littoral), the Prefecture, and the Bouches-du-Rhône hunters’ federation. In the current work, these operations were supervised by a member of the Sea, Aquatic Environments and Wetlands Department (EG), and a veterinarian. The rabbits are subsequently released into other hillside sites (Côte Bleue) to support the repopulation of hunted areas and prevent the introduction of Spanish rabbit strains. The rabbits are released, free of arthropods to avoid arthropod expansion. Furthermore, under French legislation, collecting ticks in natural environments for research purposes is not subject to any specific authorization requirement. Public participatory research programs on ticks, coordinated by French research institutions, explicitly encourage citizens to collect and submit ticks for scientific research purposes, without reference to any prior authorization requirement [12].

### Study area

**Supplementary Figure 1:**
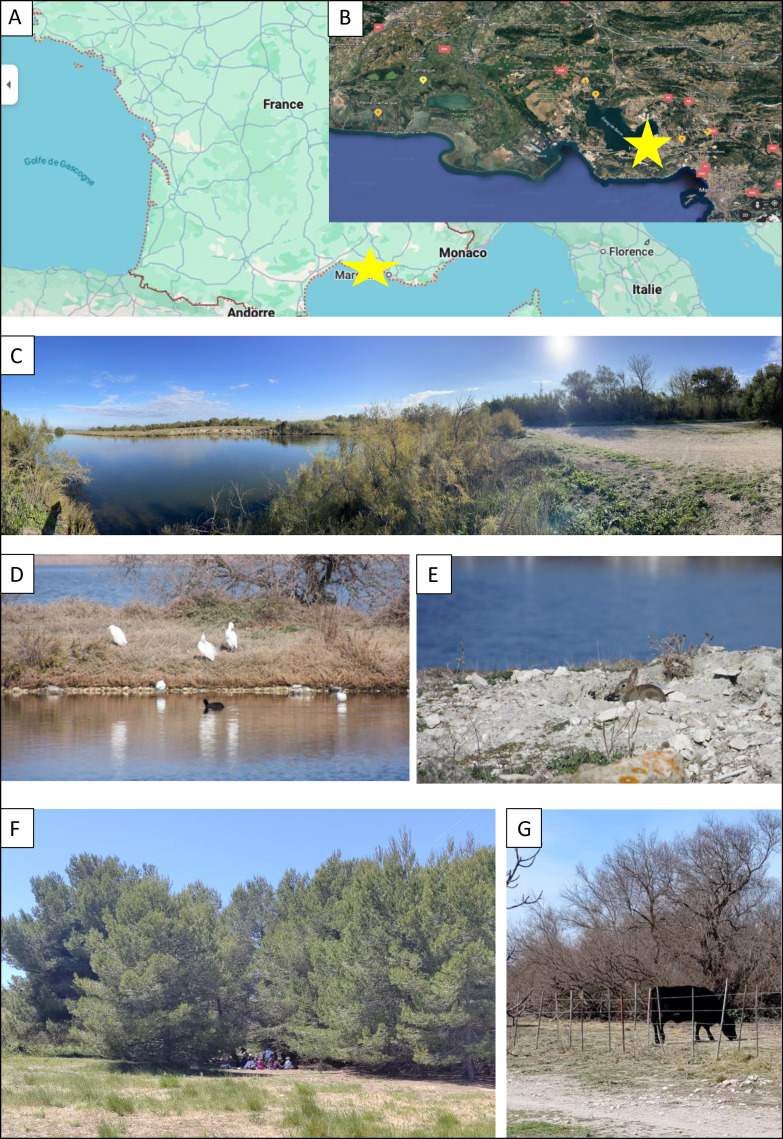
Location and landscape of the *Étang de Bolmon*, Southern France yellow star in A and B), showing the pond (C ), birds (D), rabbits (E), picknickers (F), and cows (G).

Our study was conducted at Étang de Bolmon (Bolmon pond) [13, 14, 52]. This site covers 600 ha and comprises a mosaic of natural landscapes, including a low-salinity lagoon, temporary Mediterranean marshes, wet meadows, salt marshes, steppe grasslands, woodlands (pine and riparian forests), and a dune lido. It is located near the *Salins du Lion* natural complex and is surrounded by paths designed for walkers and cyclists. Étang de Bolmon is listed as a natural zone of ecological, faunal, and floral interest; it is classified as a sensitive natural area under Article L146-6 of the Coastal Law, registered in the National Wetland Inventory, and designated as a Natura 2000 site. More specifically, the protected natural site of Étang de Bolmon has been under the land protection of the Coastal protection Agency (*Conservatoire du Littoral*), which has owned it since 1992. The area includes a discovery trail that allows visitors to explore the local flora and fauna, with interpretive panels along the way. Étang de Bolmon is well known as an excellent site for walking and nature observation (Supplementary Figure 1). The area is used year-round, with increased frequentation during periods of favorable weather.

A nature protection and environmental education association specializing in entomology and active in this area alerted our teams to unusual “giant tick” attacks on walkers and picnickers. Investigations were therefore conducted in the specific area where these incidents were reported (43° 24′ 47″ N, 5° 10′ 41″ E).

### Field capture of ticks

Tick sampling was conducted between July 2022 and September 2025, with a total of 24 field surveys performed during this period (Table 1). Sampling was carried out at variable frequencies until June 2024, followed by bi-monthly surveys until August 2024, and monthly surveys from September 2024 to September 2025.

**Table 1:** Field collection of ticks at Étang de Bolmon, southern France (2022–2025) and their morphological identification. A timeline can be seen as Supplementary Figure 3.

Collection year	Collection date	Number of collectors /duration of collection	*H. lusitanicum* (F, N)	*R. pusillus* (F, N)	*H. hispanica* (F, N)	*I. ventalloi* (F, N)	Number of ticks collected/person/hour
2022	05/07/2022	6/2 h	203 (143F)				17
	13/08/2022	2/2 h	36 (19F)				9
	21/02/2023*	3		82 (36F)	5 (0)	1(1N)	
2023	03/03/2023*	3		78 (28F)	7 (1F)		
	30/03/2023*	2	1(0)	150 (59F)			
	26/07/2023	2/2 h	120 (86F)				30
	11/06/2024	2/2 h	4 (2F)	1 (0)			1
	27/06/2024	6/2 h	47 (28F)	18 (12F)			5
	09/07/2024	1/2 h	13 (10F)				7
	25/07/2024	3/2 h	36 (15F)	1 (1F)			6
2024	08/08/2024	4/2 h	23 (18F)				3
	22/08/2024	2/2 h	37 (18F)				9
	19/09/2024	4/2 h	146 (82F)				18
	22/10/2024	3/2 h	39 (23F)				7
	07/11/2024	3/2 h	7 (4F)				1
	28/01/2025	4/2 h	27 (17F)			1(1F)	4
	27/02/2025	7/2 h	277 (130F)	62 (29F)			24
	20/03/2025	7/2 h	311 (93F)	136 (88F)			32
	24/04/2025	4/2 h	103 (65F)	6 (0)			14
2025	26/05/2025	3/2 h	131 (66F)	13 (8F)			24
	26/06/2025	5/2 h	83 (65F)	87 (49F)			17
	30/07/2025	5/2 h	160 (72F, 6 N)	11 (6F, 1N)			17
	19/08/2025	4/2 h	317 (182F, 3 N)	1 (0F, 1N)			40
	23/09/2025	4/2 h	158 (88F)				20
	**Total**	85/42 h	2279 (1226, 9 N)	647 (316F, 2N)	12 (1F)	2 (1F, 1N)	15

*Collection during ferreting operations; F, Female; N, Nymph.

**Figure 1:**
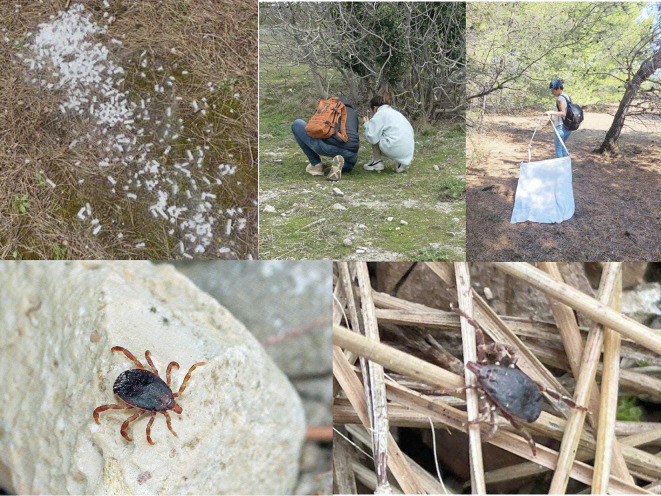
Field collection sites for ticks at Étang de Bolmon using flagging (A), dry ice placed on the ground to attract ticks (B), and direct visual searches by collectors (C). (D, E) Male *Hyalomma lusitanicum* ticks on the ground. See supplementary videos illustrating tick collection at: https://doi.org/10.5281/zenodo.20141476.

Ticks were collected during field trips using several methods, including direct visual searches by collectors walking through the area to spot ticks on the ground, and the use of dry ice to attract actively host-seeking ticks through the release of CO_2_ during sublimation. During the first sampling sessions, flagging was also performed over random 10 m transects. This consisted of dragging a piece of fabric – such as a small blanket – across vegetation behind the observer, allowing questing ticks to attach to the cloth as it passed (Fig. 1) [39, 40]. As soon as ticks were observed, they were collected manually by team members searching for active ticks on the ground. The number of collectors per field session ranged from three to six, with each team collecting for 60–90 min. See supplementary videos illustrating tick collection at: https://doi.org/10.5281/zenodo.20141476.

**Supplementary Figure 2:**
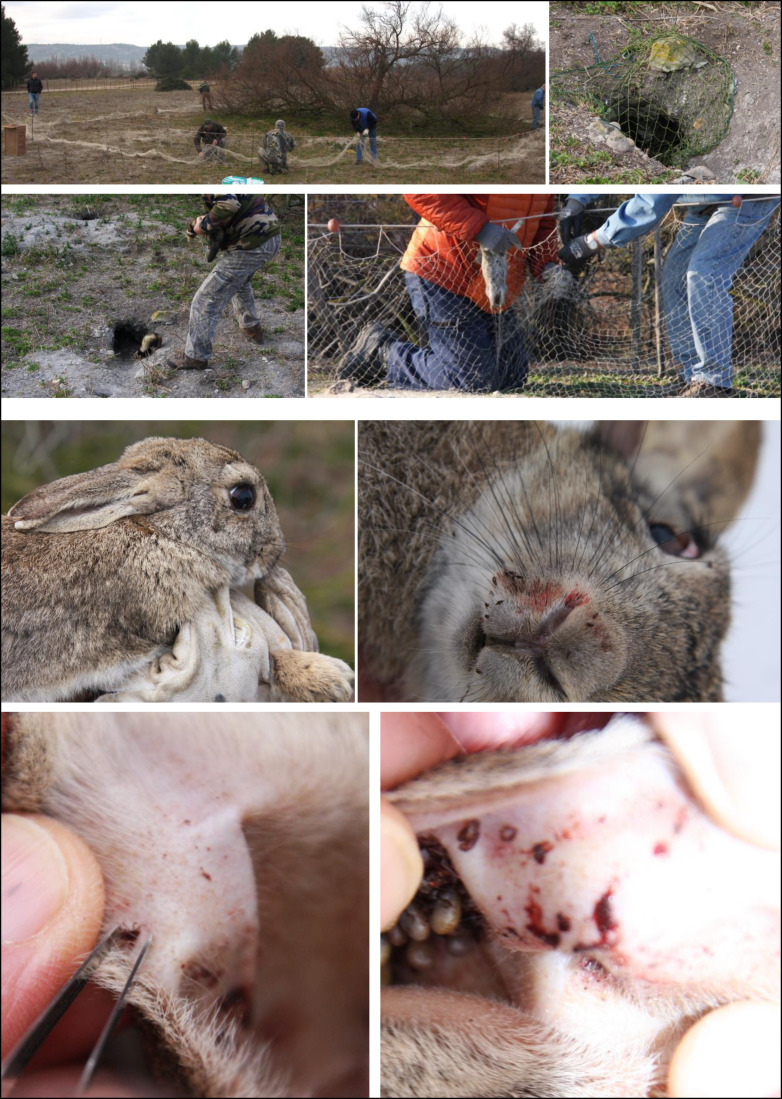
Tick collection on European rabbits during ferreting operations at *Étang de Bolmon*, 2023, Southern France.

Fieldwork also included participation in three ferreting operations in February and March 2023 – a traditional method of rabbit control. The goal of ferreting is to drive rabbits out of their burrows using trained ferrets (*Mustela putorius*), small and slender carnivorous mammals that enter the burrows and chase rabbits into nets placed at the exits. Ticks were collected from rabbits using tweezers (Supplementary Figure 2). Falcon tubes containing ticks were transported to the laboratory and stored at –20 °C after the ticks had been counted and morphologically identified. The rabbits were maintained by hunters as part of authorized hunting activities. Many fleas were also collected on this occasion (to be reported elsewhere).

### Morphological and molecular identification of ticks

All collected ticks were first morphologically identified using reference documents [50], under a Leica EZ4 stereomicroscope (Leica Microsystems, Nanterre, France) and a Zeiss Axio Zoom V16 microscope (Zeiss, Marly-le-Roi, France). Ticks were sorted by species and sex subsequently stored at −20 °C until further analyses. A subset of specimens from each morphologically identified species was randomly selected for molecular confirmation by amplification of a 465 bp fragment of the tick 16S rRNA gene, as previously described [17].

Each selected tick was bisected longitudinally. Genomic DNA was extracted individually from tick halves and stored at −20 °C until use, as previously described [17, 46]. DNA samples were then subjected to standard PCR using an automated thermal cycler [17]. DNA extracted from *R. sanguineus sensu stricto* (temperate lineage) specimens from our laboratory colonies served as a positive control, while the PCR mix without template DNA was used as a negative control. Following visualization on agarose gel electrophoresis, positive PCR products were purified and sequenced as described previously [17, 37, 41]. The obtained sequences were assembled and analyzed using ChromasPro software (version 1.7.7; Technelysium Pty. Ltd., Tewantin, Australia) and subsequently compared with sequences available in GenBank using BLAST (http://blast.ncbi.nlm.nih.gov/).

### Identification of ticks by MALDI-TOF MS

The four legs on the same side of each tick were removed, homogenized, and subjected to MALDI-TOF MS analysis with a Microflex LT mass spectrometer (Bruker Daltonics, Bremen, Germany), as previously described [35, 43, 60]. The quality and reproducibility of MS profiles obtained from tick legs – defined by the absence of background noise and peak intensities exceeding 3,000 arbitrary units – were visually assessed using FlexAnalysis v3.3 software (Bruker Daltonics, Bremen, Germany). Only spectra fulfilling these criteria were selected for further analysis. The reproducibility and species-specificity of MS spectra were evaluated using cluster analysis (MSP dendrogram). The dendrogram was generated by clustering specimens based on comparisons of their protein mass profiles (i.e., mass signals and intensities). Based on the consistency between morphological and molecular identification, MALDI-TOF MS spectra from reference specimens of each tick species were incorporated into our in-house arthropod MALDI-TOF MS reference spectra database. Blind tests were subsequently performed by matching the MS spectra of tick legs from remaining specimens against the updated reference database. The reliability of species identification was expressed by log score values (LSVs), ranging from 0 to 3, generated by MALDI-Biotyper v3.0 software. Each LSV corresponded to the degree of similarity between the MS spectra of tested samples and those of reference spectra in the database [35, 43, 60].

### DNA extraction and microorganism screening

DNA was individually extracted from one half of each tick, randomly selected from ticks collected during each sampling period between July 5, 2022 and August 22, 2025, as described above. The extracted DNA was screened by quantitative PCR (qPCR) to detect *Bartonella* spp., *Borrelia* spp., *Coxiella burnetii*, members of the *Anaplasmataceae* family, and *Rickettsia* spp., using primers and probes previously described [16, 45, 46]. DNA from ticks collected between July 5, 2022 and August 8, 2024 was tested individually, whereas DNA from ticks collected between August 2024 and May 2025 was tested in pools of 4–5 specimens. This pooling strategy was implemented to manage the large number of collected ticks, reduce reagent costs, and accelerate testing without compromising detection efficiency. qPCR assays were performed under conditions identical to those previously described [66], and positive controls were included in all runs.

DNA extracted from reference strains (*Rickettsia montanensis*, *Bartonella elizabethae*, *Anaplasma phagocytophilum*, *C. burnetii*, and *Borrelia crocidurae*) served as specific positive controls. For pooled tick DNA samples, synthetic plasmid controls containing the corresponding target sequences were used. Plasmid aliquots were prepared at a concentration of 1.0 × 10^5^ copies/5 μL and included as positive controls in each assay [7, 18]. DNA extracted from *R. sanguineus s.s.* from our laboratory colonies (known to be free of the bacteria tested) served as a negative control. Samples were considered positive when the qPCR cycle threshold (Ct) value was below 36 [17].

Samples testing positive for *Rickettsia* spp. by qPCR were subsequently analyzed using a specific qPCR assay targeting a *Rickettsia sibirica mongolitimonae* gene fragment [57]. All *R. sibirica mongolitimonae*-positive samples were further subjected to conventional PCR and sequencing of the *ompA* (outer membrane protein A) and *gltA* (citrate synthase) genes, as previously described [24]. Samples that tested positive for members of the Anaplasmataceae family by qPCR, specifically those with Ct values <28, were analyzed by conventional PCR and sequencing of the *Ehrlichia* 16S rRNA gene, amplifying a 345 bp fragment specific to Anaplasmataceae bacteria [49].

Following amplification, PCR products were resolved by electrophoresis on 1.5% agarose gels stained with SYBR Safe™ (Thermo Fisher Scientific, Illkirch, France) and visualized using a ChemiDoc MP UV imager (Bio-Rad, Marnes-la-Coquette, France). PCR products were purified and sequenced using a BigDye Terminator kit (Thermo Fisher Scientific, Illkirch, France) on a Genetic Analyzer ABI PRISM 3130 (Applied Biosystems, Courtaboeuf, France). Sequence data were analyzed and assembled using ChromasPro software (version 1.34; Technelysium Pty Ltd., Tewantin, QLD, Australia) and compared with reference sequences available in GenBank using the BLAST tool, as previously described [17].

## Results

### Sampling collection and morphological identification of ticks

**Supplementary Figure 3:**
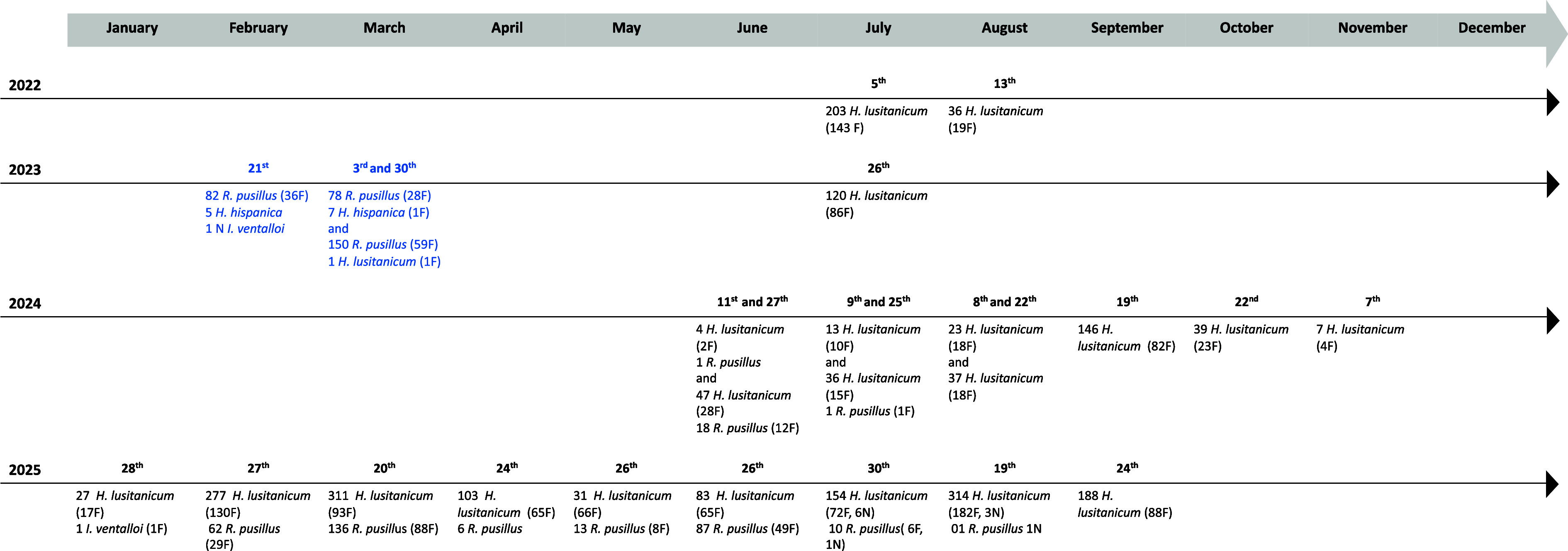
Timeline of tick collection at *Étang de Bolmon*, Southern France, 2022 - 2025. Blue characters: collection during ferreting operations.

A total of 2,940 ticks were collected during 24 field outings at the Étang de Bolmon site, including two outings in 2022, four in 2023, nine in 2024, and nine in 2025 (Table 1; Supplementary Figure 3). Among these, 1,199/2,940 (40.8%) were collected during 12 summer outings (June–August), 350/2,940 (11.9%) during four autumn outings (September–November), 455/2,940 (15.5%) during three winter outings (December–February), and 925/2,940 (31.5%) during five spring outings (March–May) (Table 1). Of the total, 2,616/2,940 (89%) ticks were collected from the ground beneath trees during 21 field outings, whereas 324/2,940 (11%) were removed from rabbits during ferreting operations (Table 1). Among the 2,616 ground-collected ticks, 956 (36.5%) were obtained during nine collections conducted without dry ice, and 1,660 (63.5%) during 12 collections using dry ice. Overall, 2,928/2,940 (99.6%) of the collected ticks were adults, including 1,544/2,928 (52.7%) females. The remaining 12/2,940 (0.4%) were nymphs. Based on morphological characteristics, ticks were identified as belonging to four species: *Hyalomma lusitanicum* (*n* = 2,279), *Rhipicephalus pusillus* (*n* = 647), *Haemaphysalis hispanica* (*n* = 12), and *Ixodes ventalloi* (*n* = 2).

All *H. lusitanicum* specimens were collected from the ground beneath trees, except for one specimen collected during a ferreting operation, but found unattached on the ground. All *H. hispanica* were collected from rabbits during ferreting operations. The *I. ventalloi* nymph was collected from a rabbit, whereas the adult was collected from the ground. Lastly, *R. pusillus* specimens were collected both from rabbits and from the ground (Tables 1, Supplementary Table 1).

### Molecular and MALDI-TOF MS identification of ticks

A total of 23 tick specimens, including 10 *H. lusitanicum*, 10 *R. pusillus*, two *H. hispanica*, and one *I. ventalloi*, were subjected to molecular identification to confirm morphological classification, using amplification and sequencing of the 16S rRNA gene. High-quality sequences were obtained for 20/23 samples (87%), including 10/10 (100%) *H. lusitanicum*, 7/10 (70%) *R. pusillus*, 2/2 (100%) *H. hispanica*, and 1/1 (100%) *I. ventalloi*. Intra-species comparison revealed 100% identity among *H. lusitanicum* sequences, 99.7–100% among *R. pusillus*, and 100% among *H. hispanica*. BLAST analysis showed that *H. lusitanicum* 16S rRNA gene sequences exhibited 98% query coverage and 99.75% identity with the *H. lusitanicum* reference sequence from Portugal (GenBank: KU130444), and 99.50 (GenBank: LC508315) – 99.74% (GenBank: ON366981) identity with other *H. lusitanicum* sequences in GenBank.

Additionally, *R. pusillus* sequences showed 100% query coverage and 99.77% identity with an *R. pusillus* sequence from Spain (GenBank: MZ420713). *Haemaphysalis hispanica* sequences exhibited 97% query coverage and 98.7% identity with a sequence obtained from *Oryctolagus cuniculus* in Spain (GenBank: MZ420715). The *I. ventalloi* sequence displayed 100% coverage and 100% identity with a sequence obtained from a bird in Italy (GenBank: MW175443).

Sequences obtained in this study from two *H. lusitanicum*, two *R. pusillus*, two *H. hispanica*, and one *I. ventalloi* have been deposited in GenBank under accession numbers PV577800–PV577801, PV577802–PV577803, PV919885–PV919886, and PV919887, respectively.

**Figure 2:**
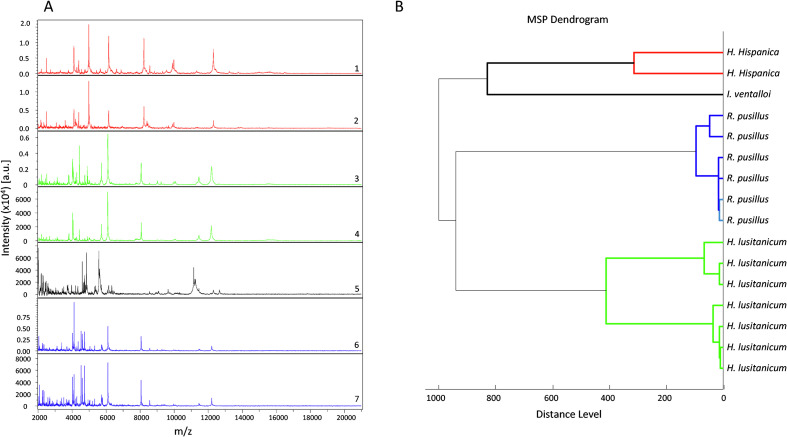
Comparison of MS spectra from four tick species collected at Étang de Bolmon (A) Representative MS spectra of *Haemaphysalis hispanica* (1–2), *Hyalomma lusitanicum* (3–4), *Ixodes ventalloi* (5), and *Rhipicephalus pusillus* (6–7). (B) Dendrogram constructed from the MS spectra of *H. hispanica, H. lusitanicum*, *I. ventalloi*, and *R. pusillus* using MALDI-Biotyper v3.3 software.

Legs from 422 *H. lusitanicum*, 349 *R. pusillus*, six *H. hispanica*, and one *I. ventalloi* were subjected to MALDI-TOF MS analysis. Mass spectra analyzed using FlexAnalysis v3.3 software showed high signal intensities (>3,000 a.u., with no background noise) in 96.4% (750/778) of tick analyses (Table 2). MS spectra visualization for one to five specimens per species demonstrated both intra-species reproducibility and inter-species specificity. These results were supported by MSP dendrograms, which showed that specimens of the same species consistently clustered on the same branch (Fig. 2).

**Table 2:** Results of MALDI-TOF MS analysis of ticks collected at Étang de Bolmon, Southern France (2022–2025).

Morphological identification (*n*)	Number tested by MALDI-TOF MS (%)	Number of good spectra (%)	Number of spectra added to MS database	MALDI-TOF MS identification species	LSVs range	LSVs ≥ 1.8 (%)
*H. lusitanicum* (2279)	422 (18.5%)	404 (95.7%)	7	*H. lusitanicum* (397)	1.70–2.49	374 (94.2%)
*R. pusillus* (647)	349 (53.9%)	339 (97.1%)	6	*R. pusillus* (333)	1.71–2.74	289 (86.7%)
*H. hispanica* (12)	6 (50%)	6 (100%)	2	*H. hispanica* (4)	1.76–2.03	3 (75%)
*I. ventalloi* (2)	1 (50%)	1(100%)	1			
**Total**	778 (26.5%)	750 (96.40%)	16		1.70–2.74	666 (90.7%)

Among the MS spectra corresponding to 20 specimens confirmed by molecular analysis, seven spectra of *H. lusitanicum*, six of *R. pusillus*, two of *H. hispanica*, and one of *I. ventalloi* were selected and added to our in-house MALDI-TOF MS reference database. The remaining 734 MS spectra – comprising 397 from *H. lusitanicum*, 333 from *R. pusillus*, and four from *H. hispanica* based on morphological identification – were blindly tested against the updated database. All 397 *H. lusitanicum* specimens were correctly identified, with log score values (LSVs) ranging from 1.70 to 2.49 (mean ± SD: 2.03 ± 0.15), and 374/397 (94.2%) achieving LSVs ≥1.8. In addition, *R. pusillus* specimens were correctly identified with LSVs between 1.71 and 2.74 (mean ± SD: 1.95 ± 0.10), and 289/333 (86.8%) achieving LSVs ≥1.8. Lastly, *H. hispanica* specimens were also correctly identified, with LSVs ranging from 1.76 to 2.03 (mean ± SD: 1.88 ± 0.07), and 3/4 (75%) achieving LSVs ≥1.8. Raw MALDI-TOF MS spectra of *H. lusitanicum*, *R. pusillus*, *H. hispanica*, and *I. ventalloi* are available at: https://doi.org/10.5281/zenodo.20187728.

### Screening for specific bacteria

DNA extracts from 1,360 *H. lusitanicum*, 454 *R. pusillus*, six *H. hispanica*, and one *I. ventalloi* were tested by qPCR either individually or in pools for the presence of microorganisms. In total, 1,062 *H. lusitanicum* and 222 *R. pusillus* specimens were combined into 214 and 45 pools, respectively.

All ticks, whether tested individually or in pools, were negative for *Borrelia* spp., *Bartonella* spp., and *Coxiella burnetii*. Two pools of *H. lusitanicum*, collected in September 2024 and February 2025, tested positive for *Rickettsia* spp., with Ct values of 34 and 35. Both pools were positive for *R. sibirica mongolitimonae* by species-specific qPCR (Ct = 31 and 34). Individual testing of the ten specimens comprising these two pools revealed four positives (Ct = 29, 32, 35, and 36). Standard PCR and sequencing of these four specimens using the *ompA* and *gltA* genes yielded two usable *ompA* sequences and one usable *gltA* sequence. BLAST analysis showed 100% coverage and 99.81% identity for *ompA* (GenBank: MF379309.1) and 100% coverage with 99.08% identity for *gltA* (GenBank: DQ423370.1). The three *R. sibirica mongolitimonae* sequences from this study were deposited in GenBank under accession numbers PX580606–PX580608.

A total of 111 pools of *H. lusitanicum* and 12 pools of *R. pusillus* tested positive for bacteria belonging to the family Anaplasmataceae, with positive *H. lusitanicum* pools detected during all sampling periods. Thirty *H. lusitanicum* specimens, grouped into six pools with Ct < 28, were selected for conventional PCR and sequencing of the Anaplasmataceae 16S rRNA gene. Usable sequences were obtained from six specimens. BLAST analysis revealed 100% coverage and 99.62–100% identity with *Candidatus* M. mitochondrii (GenBank: KX359182.1). These six *Candidatus* M. mitochondrii sequences were deposited in GenBank under accession numbers PX417374–PX417379. The 12 DNA pools of *R. pusillus* that tested positive for Anaplasmataceae had Ct values >28 and were therefore not selected for conventional PCR and sequencing.

## Discussion

In this study, we identified several tick species, including those responsible for the so-called “giant tick” attacks in a specific area of southern France, Étang de Bolmon, a popular location for walking and exploring nature. The sampling effort varied over time in both frequency and methodology, and multiple collection techniques were used. This heterogeneity in field methods likely introduced some bias in species representation and life-stage distribution. However, despite these limitations, our study yielded unique results, addressing citizen reports of attacks by unusually large ticks that generated media attention, as well as enabling the rediscovery of *H. lusitanicum* and the characterization of microorganisms associated with ticks from the Étang de Bolmon area.

Tick identification was initially performed using morphological criteria. Although unambiguous diagnostic features were observed in both males and females, species identification was definitively confirmed using molecular tools, which have been routinely employed in entomological research for the past two decades [71]. We also used MALDI-TOF MS, a proteomic tool that has recently emerged in the field of entomology, providing an opportunity to further assess its robustness for arthropod identification. Initially developed for clinical laboratories, MALDI-TOF MS was later applied to the diagnosis of genetic diseases and microorganisms, and it has recently gained prominence in entomology [65].

Previous studies by our group have detailed this approach for identifying both field-collected and laboratory-reared arthropods. LSVs greater than 1.8 are considered reliable for species identification, according to previous studies [43, 65]. Despite its advantages, MALDI-TOF MS does have limitations. Accurate identification requires comparison with reference spectra from specimens definitively identified by morphological or molecular methods. Preservation techniques can also influence protein spectra, and spectral variations may occur depending on the geographic origin of specimens [65]. Therefore, maintaining an up-to-date reference database by regularly adding new specimens is essential to ensure accurate identifications [65]. The present work provides further evidence of the reliability of MALDI-TOF MS, which was evaluated on a subset of the collected ticks.

In this study, the “giant ticks” were unexpectedly identified as *H. lusitanicum* rather than *H. marginatum*, contrary to initial assumptions based on the known distribution of tick species in southern France along the Mediterranean coast, as well as recent media coverage and public health concerns regarding *H. marginatum* and its potential role in transmitting the Crimean-Congo hemorrhagic fever virus [3].

**Figure 3:**
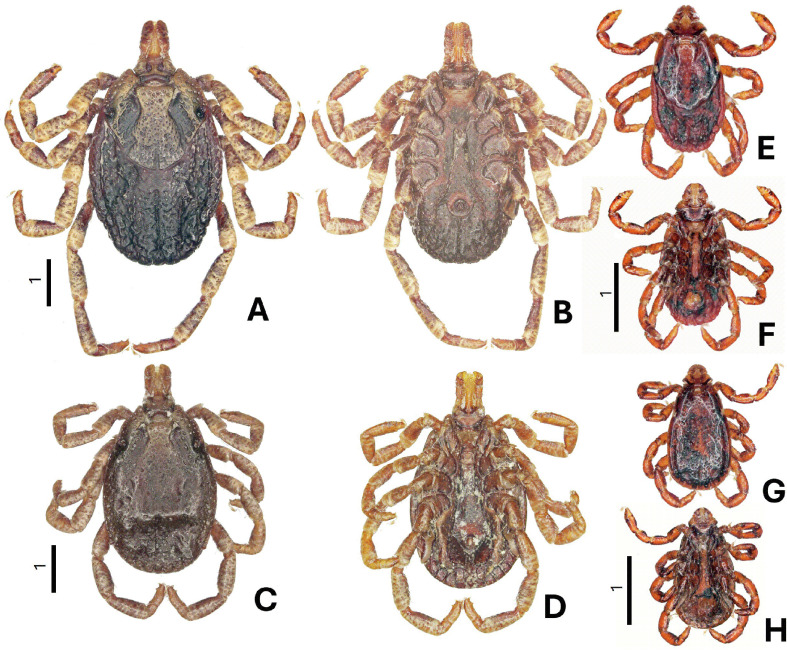
Representative images of *Hyalomma lusitanicum* (A, female dorsal view; B, female ventral view; C, male dorsal view; D, male ventral view) and *Rhipicephalus pusillus* (E, female dorsal view; F, female ventral view; G, male dorsal view; H, male ventral view) specimens collected in this study conducted at Étang de Bolmon*,* Southern France. Scale bar: 1 mm.

*Hyalomma lusitanicum* are indeed large ticks, as unfed males and females measure 4–6 mm in length and possess a long capitulum (Fig. 3). They also actively host-seek and are attracted by carbon dioxide emitted through host respiration and other odors, which explains the effectiveness of dry ice traps in their capture [48]. The distribution of *H. lusitanicum* is typically restricted to the xeric or warm scrublands of the western Mediterranean basin (Portugal, Spain, Sicily, North Africa) [63]. The species is adapted to mesomediterranean vegetation and is frequently found within rock crevices, vegetal litter, or rabbit burrows. Also, *H. lusitanicum* is a three-host tick with selective parasitic behavior: its distribution is closely linked to that of the wild rabbit (*O. cuniculus*), on which immatures feed, while adults mainly infest ungulates and occasionally carnivores. Its immature stages develop in rabbit burrows, where microclimatic conditions are favorable. However, immatures may also feed on birds. Consequently, the tick may be abundant in areas hosting both rabbits and ungulates, such as cattle, wild boar, and deer [53, 69]. Although more regular sampling would have been required, our data suggest that the peak of adult activity occurs in late spring/early summer, which is consistent with the literature. Importantly, larvae are known to be active from May to September, nymphs from July to September, and adults throughout the year, with peaks in May–July and October–November [2, 42, 68, 69]. Notably, in our study, ticks were also collected during winter.

In France, *H. lusitanicum* can be considered an emerging or re-emerging species. It has been reported sporadically in the southernmost regions, particularly in saline and alluvial habitats of the Rhône delta [50]. The species was collected until the late 1950s in Camargue, Pyrénées-Orientales, and the Var [42, 55]. Its apparent disappearance after the 1950s was attributed to myxomatosis, which decimated 90–98% of wild rabbits in France between 1952 and 1955 [25]. Later studies failed to recover *H. lusitanicum* in these areas [28, 56]. Gilot (1985) [26] suggested that the species might reappear episodically in small, localized hotspots following introductions. Given that European rabbits have been reintroduced from Spain over the past five decades [51], reintroduction of *H. lusitanicum* through parasitized hosts is plausible, though difficult to confirm. Similarly, although *H. lusitanicum* can be found on birds, their role in the dispersal of this species remains unclear [53, 69].

The *Étang de Bolmon* area offers suitable habitats for *H. lusitanicum*, although it primarily serves as a wintering site for migratory birds, which are unlikely hosts for this tick [2]. The site hosts large rabbit populations, regulated by controlled hunting, as well as occasional grazing cattle and horses. Despite its limited forest cover and ecological isolation, the site supports *H. lusitanicum* persistence. This study provides the most extensive investigation of the species’ presence in France, following sentinel alerts.

Other tick species identified, including *R. pusillus*, *H. hispanica*, and *I. ventalloi*, are rabbit-associated pholeophilous ticks adapted to burrow microhabitats. For example, *R. pusillus*, the second most prevalent species, is endophilic and monotropic, primarily infesting *O. cuniculus* [50]. It belongs to the *R. sanguineus* group and has recently been redescribed [22]. While typically restricted to rabbit burrows, *R. pusillus* can infest carnivores, domestic animals, and ungulates. Our observations of questing adults far from burrows confirm its occasional exophilous behavior, as previously noted [28].

*Haemaphysalis hispanica*, collected only during ferreting, is a small, rabbit-specific tick confined to the Mediterranean zone. After its first reports in France [42, 55], it was not detected again until the work of Gilot and colleagues [28, 50]. *Ixodes ventalloi*, also collected in this study, shares a similar ecology, being primarily associated with rabbit burrows but capable of parasitizing small mammals and birds [28].

From a public health perspective, tick-human interactions and potential pathogen transmission are key concerns. However, the mere fact of carrying a pathogen does not necessarily imply vectorial competence or the ability to transmit it.

*Hyalomma lusitanicum* can definitely bite humans, and adults exhibit active host-seeking (attacking) behavior. After the first field outings, we discontinued flagging collections because ticks were actively attacking or already visible on the ground. Regarding associated microorganisms, this tick species may be involved in the transmission of *Coxiella burnetii* to humans, the causative agent of Q fever [21]. Experimental models have suggested that this species is capable of maintaining the infection throughout its developmental cycle [34]. Among intracellular bacteria, two emerging human pathogens, *Anaplasma phagocytophilum* and *Rickettsia aeschlimannii*, have also been detected in *H. lusitanicum*. Both are known to be transmitted through tick bites [10, 68]. The presence of *Francisella tularensis*, the etiological agent of tularemia, as well as related endosymbionts (*Francisella*-*like* endosymbionts), has also been reported in this species [68]. Moreover, *C. burnetii* DNA was detected in wild lagomorphs in Spain, as well as in ticks collected from these animals, including *H. hispanica*, *R. pusillus*, and *H. lusitanicum* [6].

In a recent survey conducted in southern Spain to identify tick species parasitizing wild lagomorphs, several rickettsial pathogens (including *Rickettsia massiliae*, *R. sibirica mongolitimonae*, *R. slovaca*, and *R. africae*) were detected in *H. lusitanicum* ticks [58]. Several viruses have also been detected in this species, including the Crimean-Congo hemorrhagic fever virus (CCHFV), hepatitis E virus (HEV), and myxoma virus [68]. Regarding protozoa, the transmission capacity of *Theileria annulata* has long been suggested [33, 70], while *Theileria equi* and *Babesia pecorum* have also been detected, further emphasizing the veterinary importance of this tick [38, 68].

In the present study, we detected *R. sibirica mongolitimonae* in several *H. lusitanicum* specimens. This finding is particularly relevant for deciphering the ecology of this emerging human pathogen. *Rickettsia sibirica mongolitimonae* is a spotted fever group (SFG) *Rickettsia* first isolated from *H. asiaticum* ticks collected in Inner Mongolia, and later recognized as a human pathogen in France in 1996 [47]. According to a recent review by Santibáñez *et al.* (2025) [61], a total of 94 human cases have been reported to date, including 89 in Europe (mainly in Spain (*n* = 47), France (*n* = 36), Portugal, Greece, Türkiye, and North Macedonia), 4 in Africa, and 1 in Asia. Most cases occurred between April and July or in September. Clinical features include an inoculation eschar (sometimes multiple) at the tick bite site, a hallmark of many tick-borne SFG rickettsioses. Fever occurs in all published cases, and a generalized maculopapular rash was observed in nearly half of patients. Because a typical rope-like lymphangitis was reported in about 40% of cases, *R. sibirica mongolitimonae* infection has been referred to as lymphangitis-associated rickettsiosis.

The vector(s) of *R. sibirica mongolitimonae* have not been definitively confirmed. In Europe, this rickettsia has been detected in *H. excavatum* (Greece, Cyprus), *H. marginatum* (Spain), *R. pusillus* (Portugal, Spain, France), and *Rhipicephalus bursa* (Spain). In Turkey, it has been found in *R. bursa*, *Haemaphysalis parva*, *H. excavatum*, and *H. marginatum*. Among published human cases, four patients retained the tick responsible (one per patient), identified as female *H. marginatum*, *H. excavatum*, *Hyalomma* sp., and *R. pusillus*. Interestingly, one of the cases we previously reported occurred near *Étang de Bolmon* [67]. Another patient lived in Les Pennes-Mirabeau, an area of southern France where we have recently collected *H. lusitanicum* [57] (Stachurski *et al.* unpublished data).

In this study, we also detected *Candidatus* M. mitochondrii in *H. lusitanicum* ticks. This bacterium is an intracellular endosymbiont residing within the mitochondria of ovarian cells. It has been reported in numerous tick species worldwide, including *Ixodes holocyclus*, *Amblyomma americanum*, *Rhipicephalus sanguineus*, *H. lusitanicum*, and *Rhipicephalus microplus* [54]. It has been suggested that the abundance of “*Ca.* M. mitochondrii” in ticks may interfere with the detection of other pathogens [29].

Concerning *R. pusillus*, little is known about its affinity for biting humans. Based on years of field experience and an experimental attempt, Gilot reported that adult *R. pusillus* can bite and attach to humans, though attachment appears to be short-lived; no data exist for immature stages [27]. In our own investigations, we studied several tick-borne diseases in a household where *R. pusillus* was likely introduced by the family’s cat [19]. In the present work, we found evidence suggesting that *R. pusillus* can also bite humans, as some specimens were found on our clothing and footwear during fieldwork.

The role of *R. pusillus* as a vector of microorganisms remains poorly understood [64]. Among bacteria, *Ehrlichia* spp. and *R. massiliae* have been detected, the latter being recognized as an emerging zoonotic agent [58, 59]. Portuguese studies have reported the detection of *R. sibirica mongolitimonae* DNA in *R. pusillus* collected from a dead African mongoose (*Herpestes ichneumon*), alongside a serologically confirmed human case of infection in Portugal [15]. In 2013, we reported a cluster of one confirmed and two probable cases of lymphangitis-associated rickettsiosis caused by *R. sibirica mongolitimonae* in southern France, linked to a cat and *R. pusillus* ticks [19]. In the wild lagomorph study cited above, *C. burnetii* DNA was also detected in *R. pusillus* [6].

Finally, little is known about the two other tick species collected in this study. Interestingly, Gilot was unable to feed *H. hispanica* on humans [27]. In the recent molecular studies cited above, *R. sibirica mongolitimonae* and *C. burnetii* were also detected in *H. hispanica* [6, 58]. Regarding the affinity of *I. ventalloi* for biting humans, Gilot reported the first two records. According to him, due to its host specificity and pholeophilous behavior, human bites are likely rare. These ticks may come into contact with humans in two main ways: either through a hunted rabbit, or via another animal, e.g., domestic (e.g., a cat) or synanthropic (e.g., a hedgehog), that has attacked a rabbit or visited its burrow. In *I. ventalloi*, several microorganisms have been identified, including the Eyach virus [9], believed to be neuropathogenic to humans [8], and *Anaplasma phagocytophilum*, detected by molecular methods [62].

## Conclusion

People visiting the Bolmon pond area should take protective measures against tick bites, including the use of protective clothing, repellents, insecticide-treated clothing and post-exposure tick checks [20]. The presence of ticks in the area could be indicated so that hikers are encouraged to check themselves for ticks at the end of their walk.

The drivers of *H. lusitanicum* introduction, establishment, and occasional disappearance in southern France are poorly understood. In addition to its documented association with rabbits, ecological factors such as temperature, vegetation structure (e.g., tree and plant composition), and soil characteristics, all of which are likely to play an important role in the ecology of *Hyalomma* spp., warrant further investigation. The role of birds also deserves to be studied.

Despite its recent occurrence, its presence remains discreet except in certain specific areas, such as the Étang de Bolmon. Interestingly, the term “giant ticks,” frequently used in the media to describe these ticks, is not a scientific designation, but rather a journalistic label intended to attract attention. Other tick species, including vector-competent ones such as *Dermacentor* spp., are also relatively large.

Regarding *R. pusillus*, since its anthropophily appears very limited – even among highly exposed individuals such as hunters or their associates handling heavily parasitized rabbits – its role as a potential vector of human diseases is likely low.

In this work, the detection of *R. sibirica mongolitimonae* in *H. lusitanicum* represents another step toward deciphering the ecology of this emerging human pathogen, which has most frequently been associated with *Hyalomma* spp. ticks and, less commonly, with *R. pusillus*. It should be noted, however, that the number of positive ticks was relatively low. An arthropod may harbor endosymbionts that are not transmitted, or may transiently carry microorganisms acquired through mixed infestations or bacteremic blood meals. The vector competence of *H. lusitanicum* remains to be definitively demonstrated, as does its potential public health importance.

According to our findings, the potential role of rabbits as reservoirs of this rickettsia warrants further investigation. Experimental feeding of infected ticks on laboratory animals should be attempted to assess whether the ticks can act as reservoirs for *R. sibirica mongolitimonae* through transovarial and transstadial transmission. To date, basic questions regarding the relationship between *R. sibirica mongolitimonae* and its tick vector(s) remain unresolved, and its life cycle, like those of many spotted fever group (SFG) rickettsioses, is still incompletely understood.

As a reference center for tick-borne and rickettsial diseases, we first focused on the detection of bacteria within our area of expertise. The results of our qPCR analyses did not yet allow us to fully address all questions regarding the public health relevance of these ticks, and further work is required. In recent years, metagenomic approaches have been increasingly used to study microorganisms carried by ticks [36, 44], including *H. marginatum* [31]. The next steps will be to investigate the virome and microbiome repertoires of *H. lusitanicum* and *R. pusillus* from Étang de Bolmon using metagenomic strategies. Consistent with previous reports, the high abundance of an endosymbiont reported here may mask less abundant pathogens in molecular analyses, highlighting the importance of deep metagenomic approaches to accurately assess tick-borne pathogen diversity.

## Supplementary material

*Supplementary Table 1*: Detail of ticks collection on rabbits at *Étang de Bolmon*, southern France, during ferreting operations, 2023. ♂ Male / ♀ Female.
