# Supplementary material for: “Giant tick” attacks: dynamics of Hyalomma lusitanicum and detection of Rickettsia sibirica mongolitimonae in southern France
Source: Parasite. 2026 Jul 9;33:38. doi: 10.1051/parasite/2026037 (PMC13347966; doi:10.1051/parasite/2026037)
Supplement: Supplementary file 1 — Supplementary Table 1: Detail of ticks collection on rabbits at Étang de Bolmon, southern France, during ferreting operations, 2023. ♂ Male / ♀ Female. [file parasite-33-38-s1.pdf]

Ferreting operation date	Rabbits inspected		Tick species		
		Hyalomma lusitanicum	*Rhipicephalus pusillus*	*Ixodes ventalloi*	*Haemaphysalis hispanica*
February 21st, 2023	Rabbit 1		14 ♂ + 14 ♀		
	Rabbit 2		2 ♀		
	Rabbit 3		8 ♂ + 7 ♀		
	Rabbit 4		8 ♂ + 6 ♀		2 ♂
	Rabbit 5		8 ♂ + 1 ♀		
	Rabbit 6		8 ♂ + 6 ♀	1 Nymph	3 ♂
March 03rd, 2023	Rabbit 1		1 ♂ + 3 ♀		1 ♂
	Rabbit 2		6 ♂		1 ♂
	Rabbit 3		1 ♂ +2 ♀		1 ♂
	Rabbit 4		4 ♂ + 6 ♀		2 ♂
	Rabbit 5		4 ♂		
	Rabbit 6		1 ♂ + 2 ♀		
	Rabbit 7		8 ♂ + 9 ♀		
	Rabbit 8		10 ♂ + 1♀		
	Rabbit 9		1 ♀		1 ♂ +1 ♀
	Rabbit 10		1 ♂ + 1♀		
	Rabbit 11		2 ♀		
	Rabbit 12		2 ♂ + 1♀		
	Rabbit 13		1 ♀		
	Rabbit 14		3 ♂		
	Rabbit 15		4 ♂		
	Rabbit 16		1 ♂ + 1 ♀		
	Rabbit 17		1 ♂ + 1♀		
March 30th, 2023	Rabbit 1	1 ♂	4 ♂ + 1♀		
	Rabbit 2		7 ♂ +3 ♀		
	Rabbit 3		4 ♂ + 2 ♀		
	Rabbit 4		14 ♂ + 8 ♀		
	Rabbit 5		5 ♂ + 2 ♀		
	Rabbit 6		5 ♂ + 3 ♀		
	Rabbit 7		5 ♂ + 6 ♀		
	Rabbit 8		2 ♂		
	Rabbit 9		2 ♂ + 4 ♀		
	Rabbit 10		6 ♂ + 3 ♀		
	Rabbit 11		4 ♂ + 2 ♀		
	Rabbit 12		5 ♂ +9 ♀		
	Rabbit 13		4 ♂		
	Rabbit 14		5 ♂ + 4 ♀		
	Rabbit 15		1 ♂		
	Rabbit 16		3 ♀		
	Rabbit 17		6 ♂ + 2 ♀		
	Rabbit 18		5 ♂ + 4 ♀		
	Rabbit 19		7 ♂ + 3 ♀		
Total	42	1	310	1	12
